# Evaluation of the Radiographic Risk Factors of Postoperative Shoulder Imbalance in Adult Scoliosis

**DOI:** 10.3389/fsurg.2022.885949

**Published:** 2022-06-09

**Authors:** Wencan Ke, Bingjin Wang, Wenbin Hua, Kun Wang, Shuai Li, Cao Yang

**Affiliations:** Department of Orthopaedics, Union Hospital, Tongji Medical College, Huazhong University of Science and Technology, Wuhan, China

**Keywords:** adult scoliosis, correction surgery, postoperative shoulder imbalance, upper instrumented vertebra (UIV), radiographic shoulder height

## Abstract

**Objective:**

This study aimed to evaluate the radiographic risk factors of postoperative shoulder imbalance (PSI) after adult scoliosis (AS) correction surgery.

**Methods:**

Seventy-nine patients with AS undergoing correction surgery at a single institution were reviewed. The mean follow-up was 28 months. Patients were divided into two groups based on their radiographic shoulder height (RSH): (1) the balanced group (RSH <10 mm) and (2) the unbalanced group (RSH ≥10 mm). The preoperative and postoperative Cobb angles of the proximal thoracic (PT), main thoracic (MT), thoracolumbar/lumbar (TL/L) and upper instrumented vertebra (UIV) were measured.

**Results:**

No significant difference was found between the balanced and unbalanced groups when the UIV was T1–2, T3–4, or below T4. Univariate analysis indicated that the unbalanced group had significantly higher postoperative RSH, lower percentage PT correction, and greater percentage MT correction. The classification and regression tree analysis revealed that when the correction percentage of PT curve was more than 55.3%, 84.4% of patients acquired shoulder balance. However, when the correction percentage of PT curve was less than 55.3%, and the correction percentage of MT curve was more than 56%, 65.7% of the patients developed PSI.

**Conclusions:**

In AS correction surgery, a lower percentage correction of the PT curve and greater percentage correction of the MT curve were independent radiographic risk factors of PSI, regardless of the UIV level. Sufficient PT correction is required to achieve postoperative shoulder balance in AS correction surgery when the MT curve is overcorrected.

## Introduction

Adult scoliosis (AS) is defined as a three-dimensional deformity of the spine in a skeletally mature patient. According to previous epidemiological studies, the incidence of AS has been 17.0%–29.4% in the past decade (1, 2). As the ageing of populations in modern society accelerates, AS is becoming increasingly burdening (3). Currently, correction surgery is the only effective treatment for AS patients with a large magnitude curve (4, 5). Postoperative shoulder imbalance (PSI) is a common complications of AS correction surgery, which considerably impacts the postoperative satisfaction of patients (6). However, achieving postoperative shoulder balance remains challenging, with the total incidence of PSI ranging from 25% to 57% (6, 7). Identifying the independent risk factors of PSI can enhance our understanding of this phenomenon and aid in reducing its incidence.

Previous studies regarding the risk factors of PSI mainly focused on adolescent idiopathic scoliosis (AIS). The selection of upper-instrumented vertebra (UIV) is considered one of the main factors responsible for postoperative shoulder balance (8). Previous investigation found that a proximal UIV can avoid the occurrence of PSI (9). However, recent studies have indicated that PSI is not affected by the UIV level (10, 11). Andy et al. reported that a higher preoperative Cobb angle and increased surgical correction lead to an increased risk of PSI (10). In a retrospective review of 145 patients with AIS, John et al. indicated that overcorrection of the main thoracic (MT) curve (>54%) with less correction (<52%) of the proximal thoracic (PT) curve lead to a higher incidence of PSI, regardless of the UIV(11). However, the study of risk factors of PSI in AS correction surgery has not been reported.

AS is a progressive spine deformity, which has a more severe and rigid curve. Correction surgeries for AS always require longer fusion segments, which means that achieving postoperative shoulder balance is more difficult (12–14). The purpose of this study was to evaluate the radiographic risk factors of PSI after AS correction surgery.

## Materials and Methods

### Patient Data

This was a retrospective study conducted at a single institution, and was approved by the institutional review board of our hospital (No. S0469). The study included 79 patients with AS who underwent surgical treatment at our hospital between May 2014 and May 2020. The inclusion criteria were as follows: (1) adult patients with scoliosis who underwent posterior spinal fusion and instrumentation; (2) follow-up period≥12 months; (3) adequate preoperative and postoperative radiographs of the entire spine and appearance photos. The exclusion criteria were as follows: (1) patients with postoperative severe neurological complications; (2) patients who underwent revision surgery.

### Radiographic Parameters

All patients had a minimum follow-up period of 12 months, as the literature showed that the shoulder level is stable at one year postoperatively (15, 16). Patients were divided into two groups based on their postoperative radiographic shoulder height (RSH): (1) the balanced group (RSH <10 mm) and (2) the unbalanced group (RSH ≥10 mm). RSH is defined as the height difference between the right and left soft tissue shadows directly superior to the acromioclavicular joint on standing anteroposterior radiographs. PSI was defined as RSH ≥10 mm in this study, similar to previous studies (17, 18). The measurement of preoperative and postoperative RSH was completed by three researchers independently and blinded to each other. An average of the results by the three researchers was calculated and used. Intraclass correlation coefficients (ICC) were calculated to analyze measurement reliability of RSH (19). The Cobb angle of the proximal thoracic (PT), main thoracic (MT), and thoracolumbar/lumbar (TL/L) were measured pre- and postoperatively. The degree and percentage of correction of each curve were also calculated. In addition, the UIV was determined in all patients. The classification and regression tree analysis was used to identify independent drivers of PSI in multivariate analysis (20).

### Statistical Analysis

All statistical analyses were performed using SPSS (Version 22.0, SPSS, Chicago, Illinois, USA). Intraclass correlation coefficients (ICC) were calculated to analyze measurement reliability of RSH. The “model”, “type”, and “definition” selections of ICC were “Two-way mixed effects”, “Mean of k raters”, and “Consistency”, respectively. An ICC of more than 0.75 was considered as great reliability. Univariate analysis using the Student’s independent t-test and *χ*^2^ test were conducted to compare continuous and categorical variables, respectively. The classification and regression tree analysis was used to identify independent drivers of PSI in multivariate analysis. This method starts with the core node comprising of the total sample, each node is divided into two child nodes repetitively by recursive partitioning, thus creating a tree like structure. The classification trees were elaborated using the Gini splitting rule. The minimum number of patients for the parent node was set at 40, and the minimum for child nodes at 3. The maximum classification tree depth was 5. *P* < 0.05 was considered statistically significant.

## Results

In this study, 79 AS patients who underwent posterior instrumentation correction surgery were included (Table 1). Among them, 58 were female and 21 were male. The average age was 35.9 ± 12.7 years (ranging from 21 to 62 years). The mean follow-uptime was 28 months (ranging from 12 to 60 months). Overall, 48 patients had shoulder balance and 31 had shoulder imbalance at follow-up.

**Table 1:** Baselinse patient demographics.

Parameters	Balanced	Unbalanced	Total
Age (year)	35.7 ± 12.8	36.2 ± 12.4	35.9 ± 12.7
Gender			
Female	35	23	58
Male	13	8	21
PSI	48 (60.8%)	31 (39.2%)	79
Follow-up (month)	28.6	27.1	28

**Figure 1:**
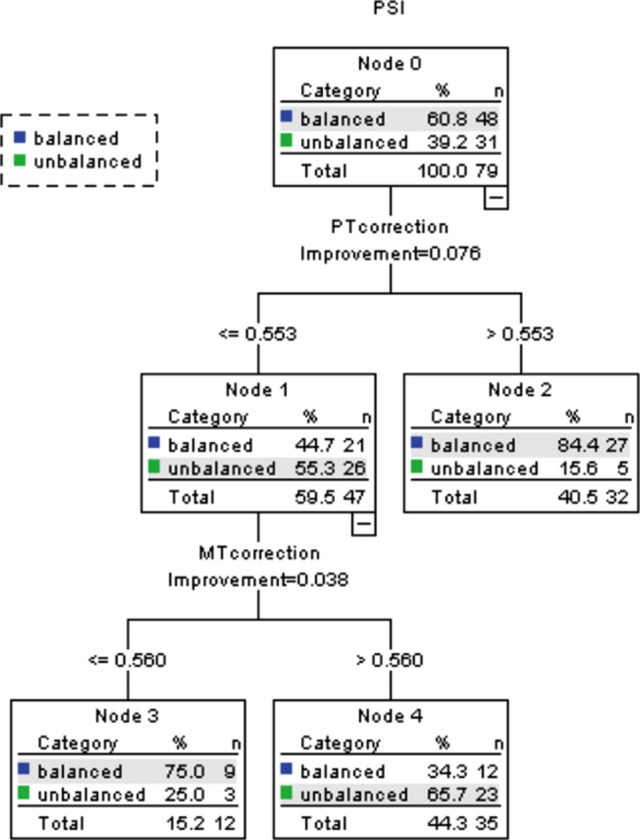
Classification and regression tree analysis for predicting postoperative shoulder imbalance.

As shown in Table 2, there was no significant difference between the balanced and unbalanced groups regarding whether the UIV was T1–2, T3–4, or below T4 (*P* = 0.512). The pre- and postoperative scoliosis parameters were shown in Table 3. The ICC for preoperative and postoperative RSH was 0.991 (95% CI, 0.987–0.994) and 0.998 (95% CI, 0.997–0.999), respectively. Univariate analysis indicated that the unbalanced group had significantly higher postoperative RSH, lower percentage PT correction, and greater percentage MT correction. The classification and regression tree analysis demonstrated that when the correction percentage of PT curve was more than 55.3%, 84.4% of the patients had balanced shoulder (Figure 1). In addition, when the correction percentage of PT curve was less than 55.3% and the correction percentage of MT curve was less than 56%, 75% of the patients achieved postoperative shoulder balance. However, when the correction percentage of PT was less than 55.3%, and the correction percentage of MT curve was more than 56%, 65.7% of the patients developed PSI (*P* = 0.038).

**Table 2:** The UIV levels of balanced and unbalanced group.

	Balanced	Unbalanced	*P*-value
UIV			0.512
T1–2	17	12	
T3–4	28	15	
>T4	3	4	

**Table 3:** Preoperative and postoperative scoliosis parameters.

Parameter	Balanced		Unbalanced		*P*-value
	Mean	SD	Mean	SD	
RSH					
Preop (mm)	6.4	2.2	7.3	2.5	0.124
Postop (mm)	5.9	2.3	21.8	7.1	<0.001*
PT					
Preop Cobb (degrees)	38.9	15.7	41.0	16.2	0.577
Postop Cobb (degrees)	16.6	6.7	19.5	8.2	0.093
Cobb correction (degrees)	22.3	10.8	21.5	8.6	0.721
Cobb correction (percentage)	56.5%	0.07	52.8%	0.07	0.026*
MT					
Preop Cobb (degrees)	79.6	31.2	82.0	31.1	0.736
Postop Cobb (degrees)	35.1	15.3	29.8	15.7	0.146
Cobb correction (degrees)	44.5	20.5	52.2	21.7	0.116
Cobb correction (percentage)	55.6%	0.11	64.0%	0.12	0.002*
TL/L					
Preop Cobb (degrees)	48.2	16.9	43.8	17.6	0.269
Postop Cobb (degrees)	19.7	10.9	18.7	9.4	0.681
Cobb correction (degrees)	28.5	12.9	25.1	13.0	0.258
Cobb correction (percentage)	58.8%	0.16	56.1%	0.14	0.465

*RSH, radiographic shoulder height; PT, proximal thoracic; MT, major thoracic curve; TL/L, thoracolumbar/lumbar curve.*

*Statistical significance.

## Discussion

The aim of this study was to analyze the factors that predict PSI after AS correction surgery. We found that a lower percentage correction of the PT curve and greater percentage correction of the MT curve were independent radiographic risk factors of PSI. Larger correction of the MT (>56%) with a relatively lower correction of the PT (<55.3%) lead to PSI in 65.7% of the patients. In contrast, when the correction percentage of PT curve was more than 55.3%, 84.4% of the patients had balanced shoulder. In addition, the incidence of PSI was independent of the UIV level.

Achieving postoperative shoulder balance is a significant but difficult goal in correction surgery of spine deformity. The choice of UIV level is considered to be one of the main factors potentially responsible for PSI, though this is still controversial. To date, there is no consensus regarding the UIV selection in correction surgery, which is a point of contention among many spine surgeons (8, 21, 22). According to a previous guidelines for AIS, a UIV of T2 was suggested for patients with a preoperative high left shoulder, T3 for those with a balanced shoulder, T4 or below for those with a high right shoulder (9). However, Jaysson et al. found that choosing T4 as UIV was more effective to avoid PSI than either T2 or T3, regardless of which shoulder was raised preoperatively (23). Recently, several studies reported that PSI is not affected by UIV levels (10, 24). The findings of these articles were consistent with our results that UIV did not affect the incidence of PSI. Our results further suggested that a proximal UIV may not be sufficient to achieve postoperative shoulder balance; rather, adequate percentage correction of the PT is paramount in avoiding the occurrence of PSI.

**Figure 2:**
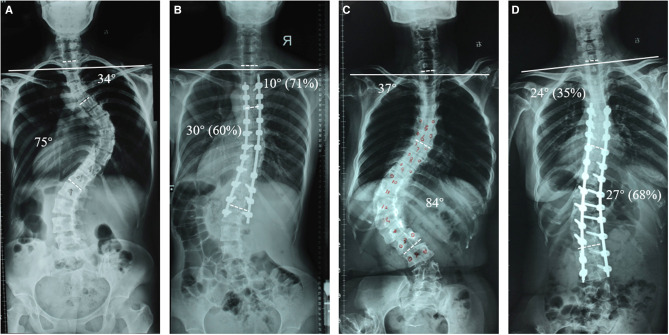
The preoperative (**A**) and postoperative (**B**) images of one patient who underwent relative larger correction of PT curve and lower correction of the MT curve, resulting in postoperative balanced shoulder. The preoperative (**C**) and postoperative (**D**) images of one patient who underwent relative lower correction of PT curve and greater correction of the MT curve, resulting in postoperative imbalanced shoulder.

Another key finding of this study was that a lower percentage correction of the PT curve and greater percentage correction of the MT curve were independent risk factors of PSI in AS correction surgery. Representative cases of a patient with postoperative balanced shoulder (relative larger correction of the PT and lower correction of the MT) and of a patient with postoperative unbalanced shoulder (relative lower correction of PT curve and greater correction of the MT curve) were shown in Figures 2A–D, respectively. In a systematic review of risk factors for PSI after correction surgery for scoliosis, Zhang et al. indicated that adequate correction of the PT and moderate correction of the MT was suggested to avoid PSI (6). Other studies also reported that overcorrection of the MT curve leads to a high incidence of PSI (24, 25). In addition, John et al. reported that larger correction of the MT curve (>54%) with simultaneous less correction (<52%) of the PT curve resulted in PSI in 59% of patients in Lenke type 1 and 2 AIS. Similar results were also observed in patients with AS in our study. Therefore, the PT curve should be sufficiently corrected to achieve postoperative shoulder balance in AS correction surgery when the MT curve is overcorrected.

To our knowledge, this is the first study to evaluate the risk factors of PSI after correction surgery of adult spine deformity. Patients with AS tend to have a larger and rigid curve, which are more difficult to correct than that in AIS. John et al. reported that when the correction percentage of PT curve was more than 52%, 80% of the patients achieved shoulder balance in Lenke type 1 and 2 AIS. However, only when the correction percentage of PT curve was more than 56% in AS correction surgery, a higher proportion of shoulder balance can be achieved. This means that a greater correction percentage of PT curve is required to maintain shoulder balance in AS. The reason may be that the PT curve in AS patients is relatively stiff, while the PT curve of AIS patients is less rigid, thus possessing self-correction ability. Indeed, several studies have reported that a flexible PT will continue to correct automatically after the MT curve is corrected (26–28). Although the PT curve is rigid in AS, the correction can be achieved through compression across the convexity and distraction through the concavity of the PT curve. During the past several decades, the posterior column osteotomy techniques have advanced considerably, thereby enabling spine surgeons to significantly correct the MT curve (29, 30). However, if the PT curve is not also adequately corrected, a higher proportion of PSI will occur.

This study has several limitations. First, this was a single-center study with a small sample size, which may result in a selection bias. Second, several other factors such as T1 tilt, clavicle angle, and coracoid height difference, were not measured and discussed. Third, only RSH was used to estimate shoulder balance in our study, which may not be fully representative of clinical shoulder balance.

## Conclusion

In conclusion, we found that a lower percentage correction of the PT curve and greater percentage correction of the MT curve were independent radiographic risk factors of PSI after AS correction surgery, regardless of the UIV level. Greater correction of the MT (>56%) with relative lower correction of the PT (<55.3%) lead to PSI in 65.7% of the patients. On the contrary, when the correction percentage of PT was more than 55.3%, 84.4% of the patients had a balanced shoulder. Therefore, the PT curve should be sufficiently corrected to achieve postoperative shoulder balance in AS correction surgery when the MT curve is overcorrected.

## Data Availability

The original contributions presented in the study are included in the article/**Supplementary Materials**, further inquiries can be directed to the corresponding author/s.
