# Supplementary material for: Evaluation of the Radiographic Risk Factors of Postoperative Shoulder Imbalance in Adult Scoliosis
Source: Front Surg. 2022 Jun 9;9:885949. doi: 10.3389/fsurg.2022.885949 (PMC9218346; doi:10.3389/fsurg.2022.885949)
Supplement: Supplementary file 1 [file Table_1_v1.docx]

| Case | Preoperative RSH | | | Postoperative RSH | | |
| --- | --- | --- | --- | --- | --- | --- |
|  | Reseacher1 | Reseacher2 | Reseacher3 | Reseacher1 | Reseacher2 | Reseacher3 |
| 1 | 10.9 | 10.3 | 10.4 | 4.3 | 4.6 | 4.7 |
| 2 | 5.5 | 5.8 | 5.8 | 2.1 | 2.4 | 2.5 |
| 3 | 8.1 | 7.5 | 7.9 | 22.1 | 22.5 | 23.4 |
| 4 | 9.2 | 9 | 8.4 | 15.2 | 15 | 16.2 |
| 5 | 9.2 | 9.4 | 8.6 | 9.3 | 9.1 | 9.6 |
| 6 | 7.5 | 7.6 | 8.2 | 28 | 30 | 27.2 |
| 7 | 10.4 | 10.5 | 11.1 | 25 | 26.6 | 24.9 |
| 8 | 6.3 | 6.8 | 7.6 | 6.2 | 5.4 | 4.4 |
| 9 | 4 | 4.6 | 3.9 | 2.1 | 2 | 2.4 |
| 10 | 8 | 7.7 | 8.6 | 6.4 | 5.5 | 4.8 |
| 11 | 7.2 | 7.3 | 7.8 | 26.7 | 26 | 27.6 |
| 12 | 8.5 | 8.2 | 7.7 | 14.1 | 13.7 | 15.5 |
| 13 | 9.1 | 9.8 | 8.4 | 5.9 | 5.6 | 5.5 |
| 13 | 6.3 | 6.4 | 7.6 | 4.1 | 4.3 | 4.6 |
| 15 | 5.6 | 6 | 6.7 | 6.5 | 6.9 | 6.6 |
| 16 | 9.6 | 10 | 10.6 | 11.3 | 12.2 | 11.8 |
| 17 | 8.6 | 8.9 | 8.1 | 22.5 | 22 | 23.2 |
| 18 | 1.3 | 1.6 | 1.7 | 27.9 | 28.7 | 26.2 |
| 19 | 8.9 | 8.8 | 7.9 | 25.8 | 27.9 | 26.3 |
| 20 | 6.2 | 6.6 | 7.4 | 6.1 | 5.2 | 4.4 |
| 21 | 6.3 | 6.7 | 7.5 | 7.2 | 8.8 | 8.2 |
| 22 | 3.9 | 4.2 | 4.4 | 4.4 | 4.7 | 4.8 |
| 23 | 4.9 | 4.1 | 4.4 | 15.7 | 16.5 | 17.2 |
| 24 | 2 | 2.3 | 2.8 | 6 | 5.8 | 5.4 |
| 25 | 7.4 | 7 | 6.8 | 22.9 | 22.8 | 22 |
| 26 | 6.9 | 7 | 7.4 | 5.2 | 5 | 5.6 |
| 27 | 6.2 | 6.2 | 6 | 8 | 7.5 | 9.1 |
| 28 | 6.5 | 6.9 | 7.1 | 9.2 | 9.4 | 9.7 |
| 29 | 8.6 | 8.5 | 9.1 | 9.4 | 8.8 | 9.7 |
| 30 | 1.4 | 1.6 | 1.9 | 14.5 | 13.6 | 16.2 |
| 31 | 7.8 | 7.9 | 7.3 | 23.4 | 24.8 | 25.7 |
| 32 | 2 | 2.5 | 1.7 | 1.8 | 2 | 2.4 |
| 33 | 6.8 | 6.7 | 7.2 | 7.2 | 6.5 | 6 |
| 34 | 10.4 | 10.5 | 11.1 | 6.6 | 6.7 | 7 |
| 35 | 3.1 | 2.8 | 3.9 | 5.4 | 6 | 4.5 |
| 36 | 6.5 | 6.8 | 7.5 | 16.2 | 15.7 | 15.5 |
| 37 | 8.6 | 8.8 | 9.2 | 29.1 | 28.8 | 28 |
| 38 | 5.5 | 5.8 | 6.9 | 5.8 | 6.7 | 7.1 |
| 39 | 6.2 | 6.7 | 6.4 | 9.5 | 9.7 | 9.8 |
| 40 | 5.5 | 5.8 | 5.9 | 4.4 | 3.6 | 5 |
| 41 | 7.3 | 7.9 | 7.4 | 32 | 32.5 | 33.5 |
| 42 | 7.4 | 7.2 | 7.8 | 33.5 | 31.6 | 32.9 |
| 43 | 10.3 | 10.3 | 10.8 | 6.1 | 5 | 4.9 |
| 44 | 7.3 | 6.7 | 6.8 | 12.2 | 13.4 | 11.8 |
| 45 | 6.2 | 6.6 | 7.4 | 2.5 | 2.3 | 2.9 |
| 46 | 5.5 | 6.3 | 5.1 | 6.5 | 5.6 | 4.9 |
| 47 | 5.6 | 6.1 | 7.2 | 4.6 | 5.3 | 6.1 |
| 48 | 5.4 | 4.6 | 5.7 | 8.2 | 7.5 | 9.1 |
| 49 | 4.2 | 4.4 | 3.8 | 7.4 | 7.9 | 6.77 |
| 50 | 6.3 | 6.2 | 6.5 | 2.4 | 2.6 | 3 |
| 51 | 7.4 | 7.7 | 8.2 | 11.4 | 12.3 | 13.7 |
| 52 | 8.3 | 8.5 | 8.8 | 12.1 | 13.6 | 11.7 |
| 53 | 6 | 6.2 | 6.9 | 5.1 | 6.3 | 5.6 |
| 54 | 5.2 | 6.3 | 5.5 | 7.5 | 8.2 | 6.7 |
| 55 | 6.7 | 6.1 | 6.5 | 5.4 | 4.5 | 6 |
| 56 | 2.9 | 3.5 | 3 | 30.6 | 30.3 | 31.4 |
| 57 | 9.1 | 9.4 | 9.6 | 26 | 26.2 | 27.2 |
| 58 | 10.5 | 10.8 | 10.9 | 2.8 | 1.9 | 3.5 |
| 59 | 5 | 5.3 | 5.4 | 6.4 | 5.6 | 4.9 |
| 60 | 4.3 | 4.4 | 4 | 2.2 | 2.5 | 2.8 |
| 61 | 2.6 | 1.9 | 2.2 | 2.2 | 2.3 | 2.5 |
| 62 | 8.2 | 9 | 9.4 | 19.8 | 20.6 | 21.2 |
| 63 | 6.2 | 6.1 | 6.4 | 8.2 | 9 | 7.5 |
| 64 | 2.2 | 2.4 | 2.7 | 7.7 | 8.2 | 9.1 |
| 65 | 7.4 | 7.6 | 8 | 12.9 | 12 | 13.4 |
| 66 | 10 | 9.7 | 10.5 | 14.1 | 14.7 | 15.7 |
| 67 | 2.4 | 2.5 | 3 | 20.4 | 20.8 | 20.2 |
| 68 | 6.2 | 6.5 | 6.7 | 5.5 | 4.9 | 6.3 |
| 69 | 10 | 9.7 | 10.6 | 9.7 | 9.5 | 10.1 |
| 70 | 6.4 | 6.1 | 6.5 | 8.5 | 8.1 | 9.4 |
| 71 | 10 | 10.4 | 10.7 | 31 | 32.4 | 30.9 |
| 72 | 8.8 | 9.2 | 8.6 | 33.6 | 32.6 | 33.8 |
| 73 | 6.9 | 7.2 | 7.7 | 21.3 | 22.7 | 23.6 |
| 74 | 2.7 | 2.4 | 2.8 | 14.3 | 15.1 | 13.7 |
| 75 | 8.9 | 9.8 | 8.5 | 9 | 9.6 | 9.4 |
| 76 | 5.2 | 5.5 | 6.3 | 3.6 | 4.5 | 5.3 |
| 77 | 6.1 | 6.7 | 6.3 | 9.2 | 9 | 9.9 |
| 78 | 7.9 | 8.6 | 8 | 7.4 | 6.7 | 5.8 |
| 79 | 5.7 | 5.4 | 5.8 | 1.9 | 2 | 2.3 |
